# Next generation sequencing and its applications in HPVassociated cancers

**DOI:** 10.18632/oncotarget.12830

**Published:** 2016-10-23

**Authors:** Musaffe Tuna, Christopher I. Amos

**Affiliations:** ^1^ Department of Biomedical Data Science, Geisel School of Medicine, Dartmouth College, Lebanon, NH, USA

**Keywords:** next-generation sequencing, genomics, omics, mutations, HPV-driven cancers

## Abstract

Approximately 18% of all human cancers have a viral etiology, and human papillomavirus (HPV) has been identified as one of the most prevalent viruses that plays causative role in nearly all cervical cancers and, in addition, in subset of head and neck, anal, penile and vulvar cancers. The recent introduction of next generation sequencing (NGS) and other omics approaches have resulted in comprehensive knowledge on the pathogenesis of HPV-driven tumors. Specifically, these approaches have provided detailed information on genomic HPV integration sites, disrupted genes and pathways, and common and distinct genetic and epigenetic alterations in different human HPV-associated cancers. This review focuses on HPV integration sites, its concomitantly disrupted genes and pathways and its functional consequences in both cervical and head and neck cancers. Integration of NGS data with other omics and clinical data is crucial to better understand the pathophysiology of each individual malignancy and, based on this, to select targets and to design effective personalized treatment options.

## INTRODUCTION

During the past three decades, it has become clear that viruses may have important player in the development of various human malignancies [1, 2]. It has been estimated that viral infections contribute to approximately 18% of all human cancers [1, 2]. Human tumor-associated viruses belong to two main families, i.e., RNA viruses (Retroviridae and Flaviviridae) and DNA viruses (Hepadnaviridae, Herpesviridae, and Papillomaviridae) [3]. DNA viruses that have been found to be associated with human malignancies include EBV (Epstein-Barr virus, Herpesviridae), HBV (Hepatitis B virus, Hepadnoviridae), HPV (Human papilloma virus, Papillomaviridae), HHV-8 (Human Herpesvirus-8, also known as Kaposi's sarcoma-associated herpesvirus, Herpesviridae), MCV (Merkel cell polyomavirus, Polyomaviridae) [4], and EVB (Erythrovirus B19, Parvovirus). RNA viruses that have been found to be correlated with human malignancies include HTLV-1 (Human T-cell leukemia virus, Retroviridae), HCV (Hepatitis C virus, Flaviviridae). HTLV-1 has been found to be associated with adult T-cell leukemia [5], HHV-8 with primary effusion lymphoma and Castleman's disease [6], MCV with Merkel cell carcinoma [4], EBV with Burkitt's lymphoma, nasopharyngeal, colorectal, gastric cancers [7–9], post-transplant lymphomas and Hodgkin's disease; HBV and HCV with hepatocellular carcinoma [10], EVB with thyroid cancer [11], HPV with head and neck squamous cell carcinoma (HNSCC), cervical, anal, penile and vulvar cancers [12]. Viruses with possible roles in human malignancies include (i) Polyomaviridae (DNA viruses), i.e., Simian virus 40 (SV40) in mesothelioma, brain and bone cancers, BK virus (BKV) in prostate and bladder cancers [13]; John Cunningham virus (JCV) in brain cancer [14], (ii) Circoviridae (DNA viruses), i.e., Tonque teno virus (TTV) in multiple myeloma, gastrointestinal, lung and breast cancers [15–17], and (iii) Retroviridae (RNA viruses), i.e., Human endogenous retroviruses (HERVs) in melanoma, germ cell, prostate, breast and ovarian cancers [18–24], and human mammary tumor virus (HMTV) in breast and endometrial cancers [25, 26].

Advances in the next-generation sequencing (NGS) technologies, including whole genome sequencing (WGS), whole exome sequencing (WES), RNA sequencing (RNA-seq), miRNA sequencing (miRNA-seq), whole genome bisulfite sequencing (WGBS), its corresponding analytical tools and ‘omics’ techniques, have provided comprehensive novel genomic and epigenomic information on HPV-associated cancers. NGS approaches have e.g. successfully been used for the identification and characterization of viral integration sites into the human genome, the identification of disrupted genes including oncogenes, tumor suppressor genes, and DNA repair genes, the detection of HPV-prone genomic instability and altered cellular pathways. In this review, we will focus on HPV-associated cancers, HPV integration sites within the human genome, and its concomitant disruption at the genomic, transcriptomic and epigenomic levels.

## HPV-ASSOCIATED CANCERS

Almost ~610,000 human cancers are the consequence of HPV infection every year in worldwide [27]. HPV infections are the primary cause of almost all cervical cancers (i.e., squamous cell cervical carcinoma and adenocarcinomas of the cervix, together 96.6%) [13, 28], a subset of HNSCC (12.9-33%) in which HPV has been found mainly in oropharyngeal tumors (50-64%), and vulvar (40-51%), vaginal (40-64%), anal (90-93%), penile (36-40%) (Table 1) [13, 29–39]. Currently, more than 200 HPV subtypes have been identified, and more than 40 of them are transmitted by sexual contact. Depending on their malignant potential, they can be classified into two groups: low-risk (LR) and high-risk (HR) HPVs [40, 41]. Twelve HPV types have been identified as belonging to the high-risk group, i.e., HPV16, HPV18, HPV31, HPV33, HPV35, HPV39, HPV45, HPV51, HPV52, HPV56, HPV58, and HPV59. HPV16 and HPV18 are predominantly (65.5% and 13.1% of the positive cases, respectively) found in cervical cancer [13, 42], whereas HPV16 and HPV33 are predominantly found in HNSCC (83.7% and 14.0% of positive cases, respectively) [13]. The occurrence of HPV in other human cancers is also rarer, such as HPV6b in bladder urothelial cancer, HPV16 in lung squamous cell carcinoma (LSCC) and uterine endometrial carcinomas (UEC) [13], and HPV16 and HPV18 in colorectal cancer (31% of colon and 0.4% in rectal cancer) [8, 43, 44], and HPV18 in gastrointestinal adenocarcinomas (19.8%) (Table 1) [8]. In the following sections, further information on the different HPV types and their respective effects on HPV-related cancers will be discussed.

**Table 1:** HPV-types infection is associated with related cancers

Cancer type	HPV type	Ref
Cervical squamous cell cancer	**16**, 18, 59, 58, 33, *11*, 31, 52, 45, *68*, 51, 56, 39, *70, 69, 68b*	[13, 56, 71]
Cervical adenocarcinoma	18, 16, 45, 33, 31	[56, 71]
HNSCC	**16, 33**, 18, 35, 56	[13, 34]
Oropharyngeal cancer	**16**, 32, 35, 45, 53, 33	[39, 58]
Vaginal carcinoma	**16**, 18, 31, 33, 45, 52, 58, **6, 11**	[35, 36]
Vulvar carcinoma	**16**, 18, 56, 31, 33, 35, 45, 51, 52, 59, *68, 58, 6, 11*	[35, 36]
Anal carcinoma	**16, 18**, *6, 11*, 31, 33,45	[36, 37]
Penile carcinoma	16, 18, *11,* 45*, 69*	[38]
Bladder urothelial carcinoma	16, *6b*	[13]
Lung squamous cell carcinoma	16	[13]
Uterine endometrioid carcinoma	16	[13]
Colon adenocarcinoma	16, 18	[8, 13]
Rectal adenocarcinoma	18	[8, 13]
Gastrointestinal adenocarcinoma	18	[8]

Bold represents the most frequent type of HPV infection; italic represents low-risk HPV’s, and the remaining HPV types are high-risk.

## HPVS AND CANCER DEVELOPMENT

**Figure 1:**
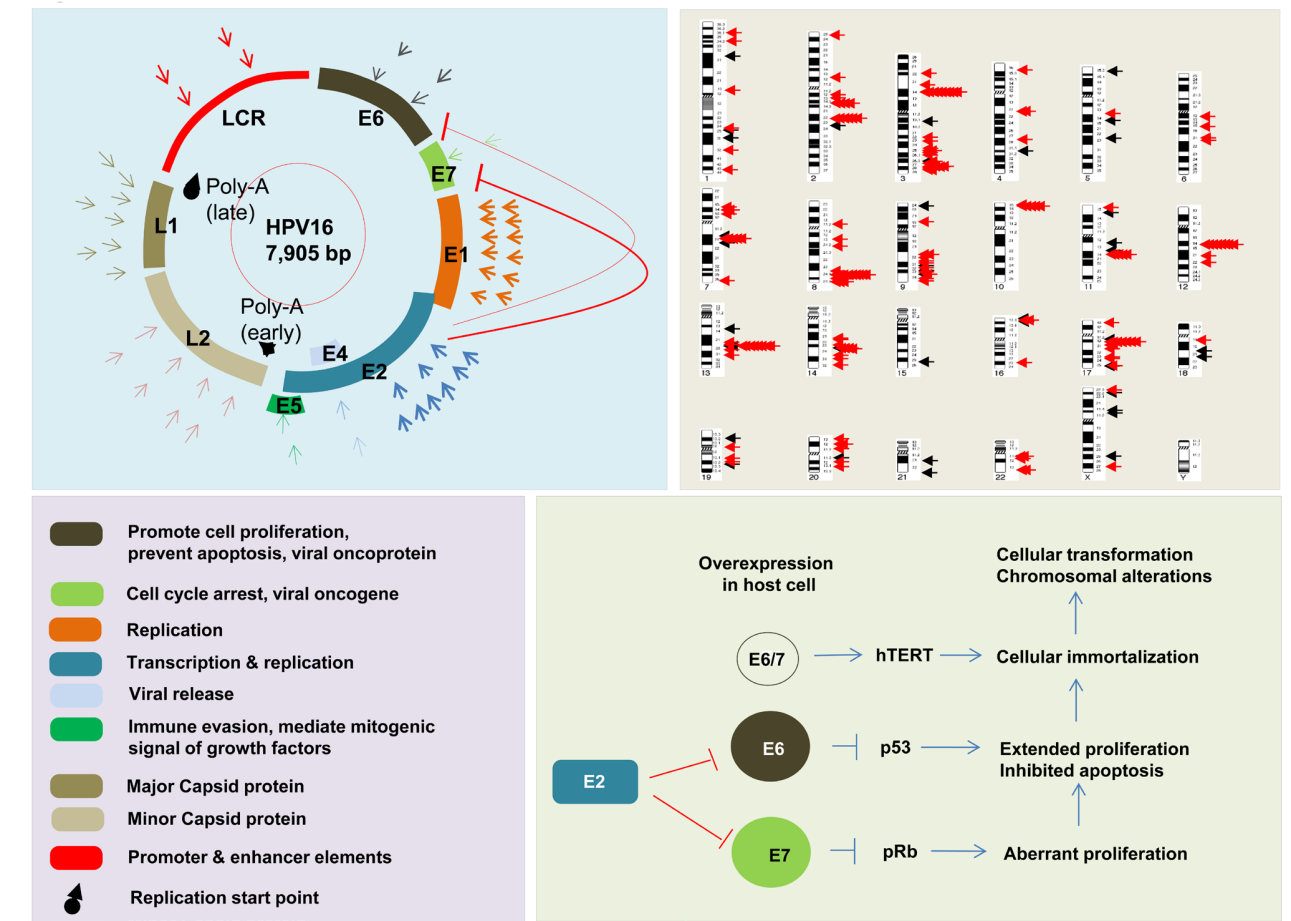
Representative figure of HPV genome, function of HPV genome components, and interaction of those components with each other in tumor development Breakpoints are distributed through the HPV genome with the most frequent in E1 in HNSC and cervical cancers. Arrows represent distribution of breakpoints in HPV genome; first circle of arrows in cervical cancer, and second circle of arrows in HNSCC (on left panel). Black mark indicates the start location for late and early replication. Distribution of HPV integration breakpoints in across the human genome is shown in upper right panel. Black arrows indicate HPV integration breakpoint in HNSCC, and red arrows indicate HPV integration breakpoints in cervical cancers. Breakpoint distribution data are based on results from TCGA, Parfenov et al, Hu et al, and Ojesina et al [34, 56, 58, 71].

The first human papillomavirus (HPV) DNA was isolated from genital warts (HPV6) and subsequently cloned by Gissmann and de Villiers in the late 1970's [45]. Shortly thereafter, the isolation of HPV16 and HPV18 DNA was reported [46]. Infection by HPVs can precipitate the development of cervical, vulvar, penile and anal intraepithelial cancers and HNSCC [47]. The HPV genome is composed of a double-stranded circular DNA that is ~8000 bp long, and contains a non-coding upstream regulatory regions (LCR), an early (E) region encompassing six open reading frames (ORFs) (*E1, E2, E4, E5, E6*, and *E7*) and late (L) region encompassing two ORFs (*L1* and *L2*) (Figure 1) [48]. The transcription of the ORFs is complex, due to the existence of multiple promoters and the formation of multiple alternative splice isoforms. Infection of the host cell by HPV activates the early promoter, which leads to expression of a polycistronic primary RNA encompassing all six early ORFs [49]. The E1 and E2 proteins are required for replication and for conserving the viral DNA as a circular episome. The viral oncogenes *E6* and *E7* provide the primary transforming activities on the HR HPVs. *E5* contributes to tumorigenesis by augmenting the role of *E6* and *E7*, while *E2* is negatively regulates their expression [50]. HR HPVs are required, but not sufficient, for the pathogenesis of anogenital and other epithelial carcinomas [51]. In early dysplastic low-grade lesions, the HR HPV genomes replicate as circular episomes (extrachromosomal circular DNA) in the normal life cycle and they retain an episomal state, while in some advanced HPV-associated cervical lesions and in most HR HPV-associated cancers the viral genome or fragments of that, integrates into the chromosomal DNA of the host cells [52, 53]. The integrated viral DNA transcripts exhibit an increased tumorigenic capacity compared to those of episomes [54, 55]. The integration rates of HPVs into the host genome are variable, depending on the tissue and HPV types involved. The HPV integration rate into the host genome is 86.5% in squamous cell carcinoma of cervix, and 53.3% in adenocarcinoma of cervix [56] and 71.2% in HPV-driven HNSCCs [57, 58]. In addition, the HPV integration rate into the host genome is 53.8% in cervical intraepithelial neoplasia and increases to 81.7% in cervical carcinomas, and by disease progression [56, 59]. Consistent with this variability, it has been found that the viral integration frequency of HPV18 is 100% and that of HPV16 is 58.5% in different HPV-related cancers [13, 60]. During the integration of HPV into the host genome, its DNA becomes fragmented. The breakpoints may occur throughout the entire HPV genome, but are mainly found in *E1* in cervical cancers [56] and HNSCC [58], and in *E2* in HNSCCs (Figure 1) [58, 61]. As a result, *E2* is either disrupted or lost. Of note, *E2* suppresses the expression of *E6* and *E7* in the episomal state. Thus, functional *E2* loss prevents suppression of the *E6* and *E7* oncogenes, consequently an increased expression of *E6* and *E7* as viral-host fusion transcripts in HPV-positive tumors which, in turn, results in increased cellular proliferation and viral immortalization [54, 55].

In most clinical samples *E6* and *E7* are found to be amplified as viral-host fusion genes. The E6 and E7 proteins inactivate the p53 and Rb tumor suppressor proteins, respectively [62, 63], thereby conferring a selective growth advantage to the cells [64–66]. E6 interacts with the host E6-associated protein and leads to p53 proteosomal degradation, which prevents apoptosis. E7 binds to the Rb protein, which releases E2F and leads to transactivation of its targets, thereby promoting cell cycle progression [65, 66]. This information indicates that integration of the HPV genome may play a crucial role in tumor development and the progression to invasive carcinoma. However, HPV integration does not always result in an increased expression of E6 and E7 oncoproteins. An increased expression of E6 and E7 oncoproteins may not sufficient for cancer development. Indeed, the expression of viral proteins may be regulated through its corresponding gene promoters as also post-transcriptionally through (host cell-derived) microRNAs such as *miR-145* [67]. *MiR-145* is the only microRNA known to directly regulate viral transcription and, thereby, its life cycle [68].

## DISRUPTION OF CELLULAR GENES THROUGH HPV INTEGRATION

Integration of a viral genome leads to disruption the host genome. Several studies have shown that half of the HPV integration sites are within or in close proximity (5Mb) of fragile sites (e.g. *FRA2K*, *FRAD3D*) [69–71], and that the remaining integration sites are within or adjacent to loci exhibiting DNA copy number alterations, including focal amplifications or deletions (e.g. *PROX1*, *FANCC*, *C9orf3*, *LINC00475*, *EGFL7*, *LOC100506190*, *RARA*, *KRT39*, *CEACAM5*, *TP63*, *ERBB2*, and *RAD51B)* or to regions exhibiting intra- or inter-chromosomal translocations, genic and miRNA regions [56, 58, 71]. It has been noted that integration occurs in regions of micro-homology among the HPVs and host genome [56, 58]. These observations suggest that HPV integration goes along with variable structural alterations within the host genome [60]. Indeed, HPV integrations may occur either within UTRs (5’ UTR: 12.6%, or 3’ UTR: 15.3%), within genic regions (exons: 2.7%, introns: 39.6%) or within intergenic regions (29.7%) [70], indicating that HPV-based disruptions may lead to the introduction of aberrant gene promoters, aberrant enhancers and/or aberrant microRNA expression patterns, thereby inactivating tumor-suppressor genes, activating proto-oncogenes, inactivating DNA repair genes and promoting genomic instability. Interestingly, different types of HPV (HPV16, HPV18 and HPV52) have been found to integrate into the intronic regions of the *RAD51B* locus in different tumors [71]. This observation supports the idea that HPV integration is a nonrandom process. Integration of HPV into the exonic or promoter regions of genes may result in their over-expression, as has e.g. been shown for *c-MYC*, *ERBB2* and *TP63*, while integration into intronic regions of genes may result in median-to-low expression, as has e.g. been shown for *RAD51B* and *FANCC*. HPV integration into the first introns of *FHIT* and *LRP1B* has been found to result in a decreased expression of these genes [56, 71]. In some HPV integrants, high expression levels were found to correlate with DNA copy number gains of loci encompassing e.g. the c-*MYC* or *ERBB2* (HPV16) genes, but this was not noted for other over-expressed genes such as *MAFA* (v-maf avian musculoaponeurotic fibrosarcoma oncogene homolog A). Moreover, in some integrants miscellaneous gene expression was found to correlate with copy number changes, including those of *TP63* and *RAD51B* in cervical cancers [71], suggesting that distinct mechanisms may underlie altered gene expression, such as gene amplification and viral promoter deregulation, in HPV-driven cancers.

Deregulation of key cellular genes by HPV integration, which may present a selective growth advantage to cells, are thought to occur through five distinctive mechanisms. A first mechanism represents integration into exon, results by truncated protein, and integration into introns, results by decreased protein [56, 58, 71]. For instance, integration of HPV into *ETS2* has been found to result in deletion of exons 7 and 8 at the integration site, resulting in a truncated form of the ETS2 protein in HNSCC [58], and integration of HPV16 into introns of *FHIT* and *LRP1B*, resulting in decreased or aberrant protein expression in cervical intraepithelial neoplasia and cervical squamous cell carcinomas [56]. A second mechanism represents amplification results with loss-of-function by integration into genes. For example, integration of HPV into *RAD51B* has been found to result in a 28-fold amplification of a 42 kb segment of intron 8 along with fragment of the viral genome, and over-expression of exons located downstream of the integrated virus, but nonfunctional protein in HNSCC [58]. Also, integration of HPV16, HPV18 and HPV52 into the same gene has also been noted in cervical cancers [71]. A third mechanism represents aberrant promoter introduction into the host gene. It has e.g. been found that the introduction of the aberrant promoter to *TP63* by HPV can cause for viral-host fusion transcript, and novel over-expressed but truncated form of p63 protein, which inhibits the action of pro-apoptotic protein [72]. A fourth mechanism represents integration of HPV within or in close proximity of a proto-oncogene or other genes, resulting in its amplification and/or over-expression. For example, HPV integration upstream of the *NR4A2* proto-oncogene has been found to result in a 248 fold amplification of a 75 kb genomic region encompassing *NR4A2* and a concomitant over-expression of this gene. Similarly, HPV18 has been found to be integrated downstream, upstream (at *POU5F1B* and *OCT4*) or within *MYC,* resulting in its amplification (20 fold) and over-expression (4.3-fold induced) in cervical cancers [56, 71, 73]. Also, integration of HPV into upstream, downstream or intronic region of *HMGA2* has been found to be resulted with its increased expression [56]. A fifth mechanism represents integration of HPV resulting in the disruption of neighboring genes by complex rearrangement (intra- and/or inter-chromosomal rearrangements). For example, amplification for certain exons of *DIAPH2,* and deletion of >100 kb segment flanking the same gene have been noted in head and neck cancer-derived UD-SCC-2 cells [70], and amplification and over-expression for complex rearrangement including *TPRG1*, *TP63* and *KLF5* have been found in HNSCC [58]. Alterations in this gene have been found to lead to chromosomal instability by misalignment of sister chromatids during mitosis [74]. In cervical cancer many HPV-integration sites have been identified, including a few hot spots in *POU5F1B* (near *c-MYC*) (9.7%), *FHIT* (8.7%), *KLF12* (7.8%), *HMGA2* 9 (7.8%), *KLF5* (6.8%), *LRP1B* (5.8%), *LEPREL1* (4.9%), *DLG2* (4.9%) and *SEMA3D* (4.9%) [56], whereas no integration hot spots (including in or near *c-MYC*) have been reported in HNSCC [34]. This observation may be due to the small number of HPV-positive HNSCCs tested and, therefore, requires evaluation in a larger cohort.

Similarly, a high viral integration rate has been found in HBV-positive tumors (76.5%), whereas none were found in HHV-positive tumors [13]. Interestingly, copy number alterations have been found near ~25% of the HBV-integration sites in hepatocellular carcinomas, with the hotspots in *TERT* locus. Overall, HBV integration has been found to be associated with copy number alterations and deregulation of gene expression in the host genome [75–77]. Also, *MLL4* and *FN1* contain hot spot integration sites for HBV in hepatocellular cancer [13], and such integration have been found to correlate with increased expression of *MLL4*, but not *FN1* [13].

## GENOMIC, EPIGENOMIC AND TRANSCRIPTOMIC LANDSCAPE OF HPV-DRIVEN CANCERS

As mentioned above, HPV integration into the host genome has been associated with deregulated gene expression, with or without copy number alterations, in cervical cancers and HNSCCs [13, 60]. HPV integration has also been found to increase the expression of E6/E7 and to induce the development of numerical and structural chromosomal alterations [78], which donate a selective growth advantage to the cells affected [55].

### DNA copy number alterations and mutations

Focal amplifications of the 2q11.2, 2q31.2 (*NFE2L2*), 7p11.2 (*EGFR*), 7q22.1, 8q24.21 (*c-MYC*), 11q13.3, 14q11.2 and 15q13.3 (*FADD*) regions are significantly higher in HPV-negative than HPV-positive HNSCCs, as well as focal deletions of the 4q35.2 (*FAT1*), 9p24.1 (*PTPRD*), 9p21.3 (*CDKN2A*), 9q34.3 (*NOTCH1*), 10q23.32 (*PTEN*), 18q21.2 (*SMAD4*) and Xp11.3 (*KDM6A*) regions [34]. In fact, both common and unique genomic alterations have been encountered in HPV-positive and negative HNSCCs. For example, focal amplifications of the 3q28 (*PIK3CA* and *TP63*) and 20q11.22 (*E2F1*) regions predominantly occur in HPV-positive HNSCCs (27.8 %), but do also occur in HPV-negative tumors (20.2%). Similarly, mutations and/or amplifications of *PIK3CA* (56%), *FGFR2* (4%) and *FGFR3* (11-14%), and deletions or truncating mutations in *TRAF3* have been observed mostly in HPV-positive HNSCCs [34, 79], whereas mutations in *PIK3CA* have also been found in HPV-negative (18.5%) cases. In addition, aberrations in the DNA repair genes *BRCA1, BRCA2, ATM, FANCG, FANCA, FANCD2* and *RAD51B* have been found to occur mostly in HPV-positive HNSCCs [34]. It has also been found that some of genes exhibit mutations only in HPV-negative HNSCCs, such as the *CDKN2A* (25.9%), *FAT1* (26.3) and *AJUBA* (7.0%) genes [34]. It is intriguing that there exists an inverse correlation between HPV status and inactivating mutations in the *CDKN2A* and *TP53* genes in HNSCCs [29, 80], i.e., mutations in *TP53* (84-87%) are more frequent in HPV-negative HNSCs than in HPV-positive (3-16%) cases [33, 34]. Of note, some tumor-suppressor genes such as *CDKN2A* have been found to be inactivated by multiple mechanisms, i.e., mutation (25.9%), deletion (homo- or heterozygous; 43.6%), abnormal splicing (2.1%) and hypermethylation (18.5%) in HPV-negative HNSCCs [34].

The mutation rate of HPV-negative tumors (4.83 mutations/megabase) has been reported to be almost twice as high as that of HPV-positive cases (2.28 mutations/megabase) [81]. Additionally, it has been found that transversions at CpG sites are more frequent in HPV-negative cases, whereas TpC mutations appear to be predominant in HPV-positive cases [34]. In fact, tobacco use is the main cause of HPV-negative HNSCCs and smoking HNSCC patients exhibit a 3.2 fold higher mutation rate than nonsmokers [81]. Consistent with this notion, genomic instability, including deletions and amplifications, are significantly higher in HPV-negative tumors than in HPV-positive cases (Table 2) [34]. Oropharyngeal squamous cell carcinomas (OSCC) are prevalent among HPV-positive HNSCCs, and are almost exclusively associated with HPV16. Similarly, *CCND1* amplifications (55%) and *CDKN2A/B* deletions (40%), and *TP53* mutations (100%) have been found to be specifically associated with HPV-negative OSCCs, similar to HNSCCs [34, 82].

**Table 2:** Genomic alterations in cervical, HPV-positive and negative HNSC carcinomas

Alterations	HPV-positive	HPV-negative	Ref
Mutation			
Cervical (squamous cell) carcinoma	*PIK3CA, PTEN, TP53, STK11, KRAS, MAPK1, HLA-B, EP300, FBXW7, NFEL2, ERBB2*		[71]
Cervical (adenocarcinoma)	*ELF3, CBFB, PIK3CA, STK11, EP300, PTEN, TP53, ERBB2, KRAS*		
HNSCC	*FBXW7, TRAF3, PIK3CA, FGFR3*	*TP53, CDKN2A, FAT1, AJUBA, CASP8, HRAS, NOTCH1, SMAD4*	[34, 82]
Amplification			
Cervical* (squamous cell carcinoma)	*MYC, ERBB2, GLI2, TNIK, NRA2, PROX1, EIF2C2, FAM179B, SERPINB4, BIRC3, YAP1, NFE2L2, TP63*		[71]
Cervical (adenocarcinoma)	*MYC, ERBB2, MCL1, ELF3, PIK3CA, SOX2*		
HNSCC	*E2F1*	*CCND1, FADD, CTTN, BIRC2, YAP1, EGFR, ERBB2, FGFR1, MYC*	[34, 82]
Deletion			
Cervical (squamous cell carcinoma)	*IDH1, PAX3, CREB1, ATM, CBL, MLL, FLI1, CDK4*		[71]
Cervical (Adenocarcinoma)			
HNSCC	*TRAF3, PTEN, CASP8*	*CDKN2A, CDKN2B, NSD1, FAT1, NOTCH1, SMAD4, Let-7c*	[34, 82]
Amplification			
Cervical (squamous cell carcinoma)	1p, 1q, 3q, 5p, 8q, 11, 14q, 17q,19q, 20p, **20q**		[71, 94]
Cervical (adenocarcinoma)	1q, 3q, 17q, 19q, 20p		[71]
HNSCC	1q25.1, 3q26.32, 3q28, 5p15.33, **11q13.3**, 12p13.31, 14q24.1, 20p11.21, 20q11.22, 21q22.3	**2q11.2, 2q31.2**, 3q26.33, 5p12, 5p15.33, **6p12.1,6q12, 7q22.1,** 8p11.23, 8q11.21, **8q24.21**, 9p13.3, 9p23, 9p24.1, 11p13, 12p13.33, 12q15, 13q22.1, **17q12**, 18p11.31, 18q11.2	[34, 70]
Loss			
Cervical (squamous cell carcinoma)	2q, 3p, 3q, 4p, 4q, 6p, 6q, 8p, 10p, 10q, 11p, 11q, **13q**, 17p,19p		[71, 94]
Cervical (adenocarcinoma)	4p, 4q, 11q, 11p, 16q, 16p, 18q, 19p		[71]
HNSCC	2q37.3, 3p13, 3p14.1, 3p24.1, 6p25.3, 6p21.33, 7q36.1, 7p22.3, 11q23.3, 11q14.2, 1314.2, 16q13, 17q25.3, 19p13.3,	1p36.13, **1p13.2**, 2q21.2, 2q36.2, 2q22.1, 3p25.3, **3p14.2**, 3p12.2, 4q22.1, **4q35.2, 5q11.2, 5q15, 5q35.3**, 6p25.3, 7q36.1, **8p23.2, 9p24.1, 9p21.3**, 9p13.1, **9q34.3**, 10p11.21, 10q23.31, 11q14.1, **11q23.1, 13q12.11**, 14q11.2, **14q32.32, 16q12.1**, 16q23.3, 17q25.3, **18q21.2**, **18q23, 19p13.3, 19q13.43**, Xp21.3, **Xp11.3, Xq21.33**,	[34, 70]

* Amplifications correlate with integration sites of HPV, bold indicates regions that are statistically significant.

Mutations in *PIK3CA* (14%), *PTEN* (6%), *STK11* (4%), *EP300* (16%), *FBXW7* (15%), *HLA-B* (9%), *MAPK1* (8%), *NFE2L2* (4%), *TP53* (9%), and *ERBB2* (5%), are common in cervical squamous cell carcinomas, while mutations in *ELF3* (13%) and *CBFB* (8%) are frequent in adenocarcinomas of the cervix. Interestingly, mutations in *PIK3CA, EP300* and *FBXW7* have also been reported in HPV-positive HNSCC [83]. The rate of somatic mutation is greater in squamous cell carcinomas than in adenocarcinomas within the Tp*C dinucleotide context [71]. Interestingly, it has been noted that DNA copy number profiles are different among HPV types in cervical cancers, i.e., DNA copy number changes are more frequent in non-HPV16 (HPV18-, HPV31-, HPV45- and HPV52-positive) than in HPV16-positive cervical cancers. In particular, DNA copy number losses are more common in non-HPV16 cases than in HPV16-positive cases. In addition, DNA copy number losses in the chromosomal regions 2q, 4p, 4q,6p, 6q, 8q and 17p (*TP53* region), and copy number gains in chromosome 1 are more common in HPV31-positive than in HPV16-positive cervical cancers, whereas DNA copy number gains in chromosome 3q (*PIK3CA* region) are more common in HPV16-positive than HPV31-positive cervical cancers [84]. These data indicate that each HPV type may induce a distinct genomic profile in cervical cancers, and thus need to be evaluated separately rather than collectively. On the other hand, it has been found that both squamous cell carcinomas and adenocarcinomas of the cervix share a copy number gain at 20q [60]. DNA copy number gains in chromosome 3q are more frequent in squamous cell carcinomas than in adenocarcinomas of cervix, whereas copy number losses in chromosome 13q are more frequent in squamous cell carcinomas of the cervix and in HNSCC [60]. This raises the question whether different HPV types induce the same or different chromosomal aberrations and/or even distinct disease subtypes. The most significant copy number alterations and mutations in cervical cancers and HNSCCs are listed in Table 2.

### Deregulated pathways

**Figure 2:**
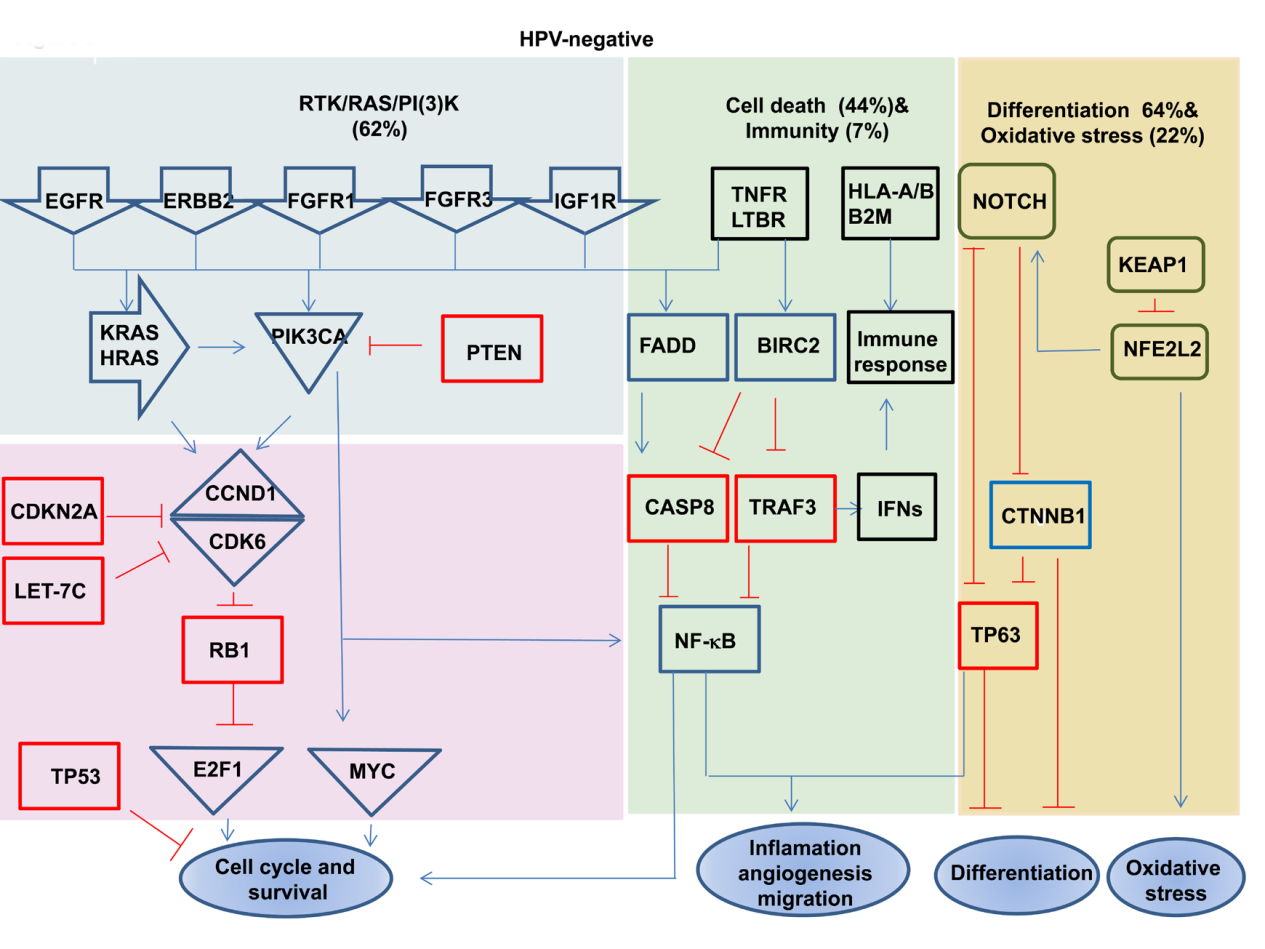
Signaling pathways deregulated in HPV-positive and negative HNSCC Red boxes highlight the tumor suppressor genes, and blue boxes highlight the oncogenes. Deletion, amplification, and somatic mutations are included in pathway alterations. Data are based on results from TCGA [34]. The present of tumors showing the indicated alterations.

HPV-driven cancers (HNSCCs and cervical cancers) show shared genomic alterations and shared deregulated cellular pathways, with different frequencies [13, 85]. For example, the Notch pathway has been found to be deregulated in both HNSCCs and cervical cancers, with *NOTCH1* mutations predominant in HPV-negative HNSCCs (20%) and infrequent in HPV-positive cases (8%). The same pathway has been found to be deregulated by mutations in other genes, such as *FBXW7*, in cervical cancers [86, 87]. In fact, even changes including mutations, deletions and amplification in the RTK-RAS-PI3K, NOTCH and NFKB cell cycle pathways, as well as other pathways, have been reported to occur in both HPV-positive and HPV-negative cases, but their rates and the genes involved in these pathways, including their activation and inactivation mechanisms, differ between the HPV-negative and HPV-positive groups. For instance, inactivation of the wild type p53 and RB1 proteins by the HPV proteins E6 and E7 that control cell cycle progression has been found to be common in HPV-positive cases, while inactivation of *TP53* by mutations is common in HPV-negative cases. Disruption of the *PIK3CA, FGFR3, TRAF3* and *E2F1* genes is predominant in HPV-positive tumors, while aberrations of the *EGFR, PTEN, FGFR1, CCND1, FADD, CASP8, FAT1, NOTCH1* and *NFE2L2* genes are more common in HPV-negative cases [34]. Consistent with this notion, it has been found that mutations and/or copy number alterations occur frequently within the PI3K signaling pathway in HPV-positive HNSCCs [*PIK3CA* (30-56%), *PTEN* (6%) and *RICTOR* (4-6%)] as well as in HPV-negative cases [*PIK3CA* (16-34%), *PTEN* (12%), *AKT* (5%), *RICTOR* (4-9%) and *mTOR* (2%)] [33, 34]. Even though the RTK-RAS-PI3K (62% in HPV-negative versus 61% in HPV-positive), cell death (44% in HPV-negative versus 31% in HPV-positive), immunity (7% in HPV-negative versus11% in HPV-positive), differentiation (64% in HPV-negative versus 44% in HPV-positive) and oxidative stress (22% in HPV-negative versus 3% in HPV-positive) pathways have been found to be deregulated in both groups with different frequencies (Figure 2), and, the prognosis has been found to be better in HPV-positive HNSCC cases [34]. Similar to HNSCCs, it has been found that PI3K-AKT pathway genes are activated by mutations in 31% of the cervical squamous carcinomas and 24% of the adeno- and adeno-squamous cases. Interestingly, mutations in the most frequently activated gene, *PIK3CA*, are more common in HPV16-positive than in HPV18- and HPV45-positive cervical cancers [88].

### Epigenetic alterations

In contrast to the genomic changes mentioned above, HPV-positive HNSCCs appear to exhibit distinct epigenetic profiles compared to HPV-negative cases [34, 58, 82, 89]. For example, the tumor-suppressor genes *BARX2* and *IRX4* have been found to be hyper-methylated and, consequently, expressed at lower levels in HPV integration-positive HNSCCs compared to integration-negative cases, whereas *SIM2* and *CTSE* genes have been found to be hypo-methylated and, consequently, expressed at higher levels in HPV integration-negative HNSCCs compared to integration-positive cases [58]. Similarly, *IRX4* has been found to be hyper-methylated and expressed at a lower level in HPV integration-positive oropharyngeal tumors compared to non-integrated tumors [90].

### Trancriptomic alterations

In concordance with the epigenetic profiles described above, gene expression array studies have revealed similarities and differences between HPV-positive and HPV-negative cancers, as well as similarities between HPV-positive HNSCCs and cervical cancers [34, 71, 85, 91]. Different genes acting in the cell cycle pathway have been found to be activated in HPV-negative HNSCCs compared to HPV-positive cases. For example, *CCND1, CCND2* and *CCNA1* were found to be up-regulated in HPV-negative HNSCC, while *CDKN2A*, *CCNE2*, *CCNB1* and *MCMs* were found to be over-expressed in HPV-positive cases. In addition, multiple genes that regulate DNA replication and cell proliferation, including *PCNA, E2Fs, CDC2,* and *CDC7*, were found to be significantly up-regulated in HPV-positive HNSCCs and cervical cancers compared to HPV-negative HNSCCs. Interestingly, three testis-specific genes *SYCP2*, *TCAM1* and *STAG3*, were found to be over-expressed in HPV-positive HNSCCs and cervical cancers. Normally, these genes are not expressed in somatic cells [85].

### Deregulated miRNAs

Based on microRNA (miRNA) profiling, HPV-positive and -negative HNSCCs can be clustered into two distinct subgroups, whereas significant similarities between HPV-positive HNSCC and cervical cancers have reported [34, 71, 91]. The expression of *miR15a* (4.19 fold) and *miR-20b* (3.42 fold) has been found to be higher in HPV-positive HNSCCs than in HPV-negative cases, while the expression of *miR-139-3p* (2.26 fold) and *miR-145* (2.19 fold), among others, has been found to be lower in HPV-positive than in HPV-negative HNSCCs. Also, the expression of *miR-145* has been found to be lower in HPV-positive cervical cancers [92] and this miRNA has been reported to suppress the growth of cervical cancer cells *in vitro* [93]. Notably, five significantly deregulated miRNAs, i.e., *miR-9* (1q23.2), *miR-15b* (3q25.32), *miR-28-5p* (3q27.3), *miR-100* and *miR-125b* (11q24.1) have been found to be associated with recurrent chromosomal alterations [92]. *MiR-9* is a potential oncogene, and its over-expression has been associated with increased copy numbers of chromosome 1q and increases cell viability, anchorage-independent growth and migration *in vitro* [94]. In concordance, the expression of *miR-34s* was found to be deregulated after HPV infection by the viral oncoprotein E6 [91]. It has also been found that the expression of miR-20b, miR-9, miR-9*, miR-492, miR-545, miR-591, miR-422a, miR-142-3p, miR-383,miR-520g, miR-101, miR-381, miR146a, miR34a, miR412, miR-155, miR-554, miR380-5p, miR-16, miR-219, miR-29c, let-7g, miR-30e-5p and *miR-139* is significantly higher in HPV-positive OSCCs than in HPV-negative cases, whereas the expression of *miR-193b, miR-107, miR-23a, miR-365, miR-452, miR-198, miR-196a, miR-181d, miR-324-5p, miR-550, miR-487b, miR-599, miR-382, miR-221* and *miR-22* is lower in HPV-positive versus HPV-negative OSCC cases [95]. Interestingly, the expression of *miR-34* has been found to be down-regulated in cervical cancers [91], while it is has been found to be over-expressed in HPV-positive oropharyngeal carcinomas [95], suggesting that expression of miR-34 may be tissue dependent, and may not be related to HPV status.

## CONCLUSION AND FUTURE DIRECTION

Recent efforts of The Cancer Genome Atlas (TCGA) network together with other NGS and ‘omics’ studies in virus-associated tumors have provided detailed information on the etiology of HPV-driven cancers. It is important to stress that integration of NGS data with other ‘omics’ data is crucial to better understand the pathophysiology of each individual disease and, based on this, to select targets and effective personalized treatment options. HPV-positive and -negative cases may exhibit both similar and different genomic alterations, whereas the epigenomic and transcriptomic profiles may be distinct between these groups. For example, the expression of cell cycle regulatory and testis-specific genes distinguishes HPV-positive from HPV-negative HNSCCs. Similarly, over-expression of *CCND1* is frequently observed in HPV-negative cases, whereas up-regulation of *E2F1* is common in HPV-positive cases. In contrast, mutation and/or amplification of *PIK3CA* are a common feature of both HPV-positive and -negative HNSCCs and cervical cancers [34]. These data indicate that for the design of combination therapies both shared alterations (in genes or pathways) between groups, and alterations that are specific to each group may be important to bear in mind. It is also clear that for selecting targets and therapeutic agents, and thus for designing more effective therapies, further interrogations are required. To this end, it is considered critical to integrate NGS and ‘omics’ data with clinical data in order to evaluate its functional consequences. In addition, both proteomics and epigenemics studies in HPV-associated cancers have so far been limited. In particular ‘omics’ studies such as glycomics and metabolomics require further interrogation. Also, the (anomalous) localization of aberrant proteins in cancer cells and its functional consequences requires further clarifications. Finally, multicenter studies may be required to increase sample size, especially for HPV-positive HNSCCs, to uncover novel biomarkers, therapeutic targets and, ultimately, potentially effective treatment options.
